# Molecular Detection of *Haplorchis pumilio* Eggs in Schoolchildren, Kome Island, Lake Victoria, Tanzania

**DOI:** 10.3201/eid2811.220653

**Published:** 2022-11

**Authors:** Hyejoo Shin, Bong-Kwang Jung, Seungwan Ryoo, Sooji Hong, Heonwoo Jeong, Hoo-Gn Jeoung, Sunhye Kim, Sun Kim, Min-Jae Kim, Hansol Park, Keeseon S. Eom, Godfrey M. Kaatano, Jong-Yil Chai

**Affiliations:** Korea Association of Health Promotion, Seoul, South Korea (H. Shin, B.-K. Jung, S. Ryoo, S. Hong, H. Jeong, H.-G. Jeoung);; Good Neighbors International, Seoul (Sunhye Kim, Sun Kim);; Asan Medical Center, University of Ulsan College of Medicine, Seoul (M.-J. Kim);; Parasite Resource Bank, Chungbuk National University School of Medicine, Cheongju, South Korea (H. Park, K.-S. Eom);; National Institute for Medical Research, Mwanza, Tanzania (G.M. Kaatano);; Seoul National University College of Medicine, Seoul (J.-Y. Chai)

**Keywords:** *Haplorchis pumilio*, molecular diagnosis, parasites, heterophyid trematodes, fishborne trematodes, Lake Victoria, Tanzania

## Abstract

A survey of intestinal helminths targeting 1,440 schoolchildren in 12 primary schools on Kome Island (Lake Victoria), Tanzania, revealed small trematode eggs in 19 children (1.3%), seemingly of a species of *Haplorchis* or *Heterophyes*. The eggs were molecularly confirmed to be *Haplorchis pumilio* on the basis of 18S and 28S rDNA sequences.

*Haplorchis pumilio*, a species of the zoonotic minute intestinal flukes belonging to the family Heterophyidae, was first discovered in the small intestines of birds and mammals in Egypt (*1*). Infection with this fluke also occurs in humans through the consumption of raw or improperly cooked fish harboring the metacercariae. Abdominal pain, diarrhea, lethargy, anorexia, malabsorption, and weight loss are the possible clinical symptoms (*2*). This fluke is widely distributed geographically from Africa to Asia, Australia, and the Americas (*1*). However, human infections were reported in only 5 countries in Africa and Asia: Egypt, China, Laos, Thailand, and Vietnam (*1*). We recently surveyed the prevalence of intestinal helminths among schoolchildren in 12 primary schools on Kome Island, Lake Victoria, Tanzania (Figure 1, panel A). We detected a low-grade prevalence of an apparent species of *Haplorchis* or *Heterophyes* by the recovery of eggs in fecal samples. We used molecular methods to confirm the eggs to be *H. pumilio* on the basis of 18S and 28S rDNA gene sequences.

**Figure 1:**
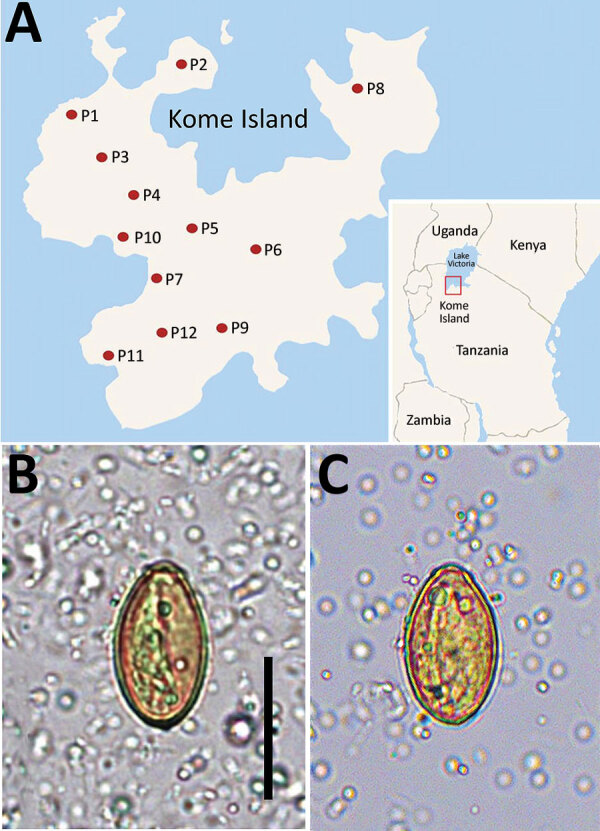
Geographic distribution and imaging from study of *Haplorchis pumilio* eggs in schoolchildren, Kome Island, Lake Victoria, Tanzania. A) Locations of 12 primary schools (P1–P12) surveyed on Kome Island. Inset shows location of Kome Island. B–C) Small trematode eggs (30.0–30.2 µm long and 16.5–16.6 µm wide) detected in schoolchildren, yellowish-brown in color, oval, and operculate with a thick shell and prominent (B) or less prominent shoulder rims (C). Scale bar = 25 µm.

## The Study

An international collaborative project between South Korea and Tanzania named “Rapid assessment of schistosomiasis and soil-transmitted helminthiases on Kome Island, Buchosa District, northwestern Tanzania” was implemented during 2020–2022. This project was approved by the Ethics Committee of the Korea Association of Health Promotion, Seoul, South Korea (IRB no. 130750-202009-HR-019). Fecal examinations were performed on 1,440 schoolchildren in 12 primary schools by using the Kato-Katz thick smear technique.

The number of overall helminth egg–positive cases was 631/1,440 (43.8%): *Schistosoma mansoni* (564 [39.2%]), *Trichuris trichiura* (42 [2.9%]), *Ancylostoma duodenale* or *Necator americanus* (27 [1.9%]), small trematode eggs (STE) (19 [1.3%]), *Enterobius vermicularis* (16 [1.1%]), and others (unidentified) (7 [0.5%]). The STE were operculate, oval, yellowish-brown in color, 29.0–31.6 (mean 30.4) µm long, and 14.8–17.6 (mean 16.5) µm wide (n = 6). They seemed to be the eggs of a *Haplorchis* or *Heterophyes* species (Figure 1, panels B and C). The STE-positive fecal samples were preserved in 100% ethanol for molecular analysis.

We extracted DNA from 20 mg of the fecal sediment by using the DNeasy Tissue and Blood kit (QIAGEN, https://www.qiagen.com) after a modified formalin-ether concentration method in which formalin was replaced with water. The sediment was washed several times with distilled water. We performed PCR targeting the 18S and 28S rDNA of *Haplorchis* species using the primers we designed on the basis of the reported nucleotide sequences of *Haplorchis* and *Heterophyes* in GenBank (Table 1). We conducted PCR in a final volume of 20 µL using 5x PCR Premix (GenomicsOne, https://www.genomicsone.kr). The procedure included an initial denaturation at 94°C for 3 min, followed by 40 cycles of denaturation at 94°C for 30 s, annealing at 60°C for 30 s, extension at 72°C for 30 s, and final extension at 72°C for 5 min. The amplicons were electrophoresed in 2.0% agarose gel, and DNA sequencing was performed using the Sanger method (*3*) by Macrogen Inc. (https://www.macrogen.com). We aligned sequences of the amplicons and generated phylogenetic trees with the maximum-likelihood method in MEGA version 7.0 software (https://www.megasoftware.net) by using the Kimura 2-parameter model with 1,000 bootstrap replications.

**Table 1:** Primers used for DNA amplification in study of *Haplorchis pumilio* eggs in schoolchildren, Kome Island, Lake Victoria, Tanzania*

Target gene	Primer	Sequence, 5′ → 3′	Length, bp
18S rDNA	18S 1F	ATACGGGACTCGTTAGAGGC	504
	18S 1R	TACAAATGCCCCCGTCTGTC	
28S rDNA	28S 1F	AGTGAACAGGGAAAAGCCCAG	897
	28S 1R	TCAGGTGGAAAGTCTACCGC	
	28S 2F	ATAGCGAACAAGTACCGTGAGG	
	28S 2R	ACATGTTACTCTCCTTGGTCCG	659

*Primers were designed using Geneious primer design software (https://www.geneious.com) based on the 18S rDNA sequences of *Haplorchis pumilio* (GenBank accession no. AY245706) and *H. taichui* (accession no. AY245705) and the 28S rDNA sequences of *H. pumilio* (accession no. HM004173) and *Heterophyes heterophyes* (accession no. KU559553).

**Figure 2:**
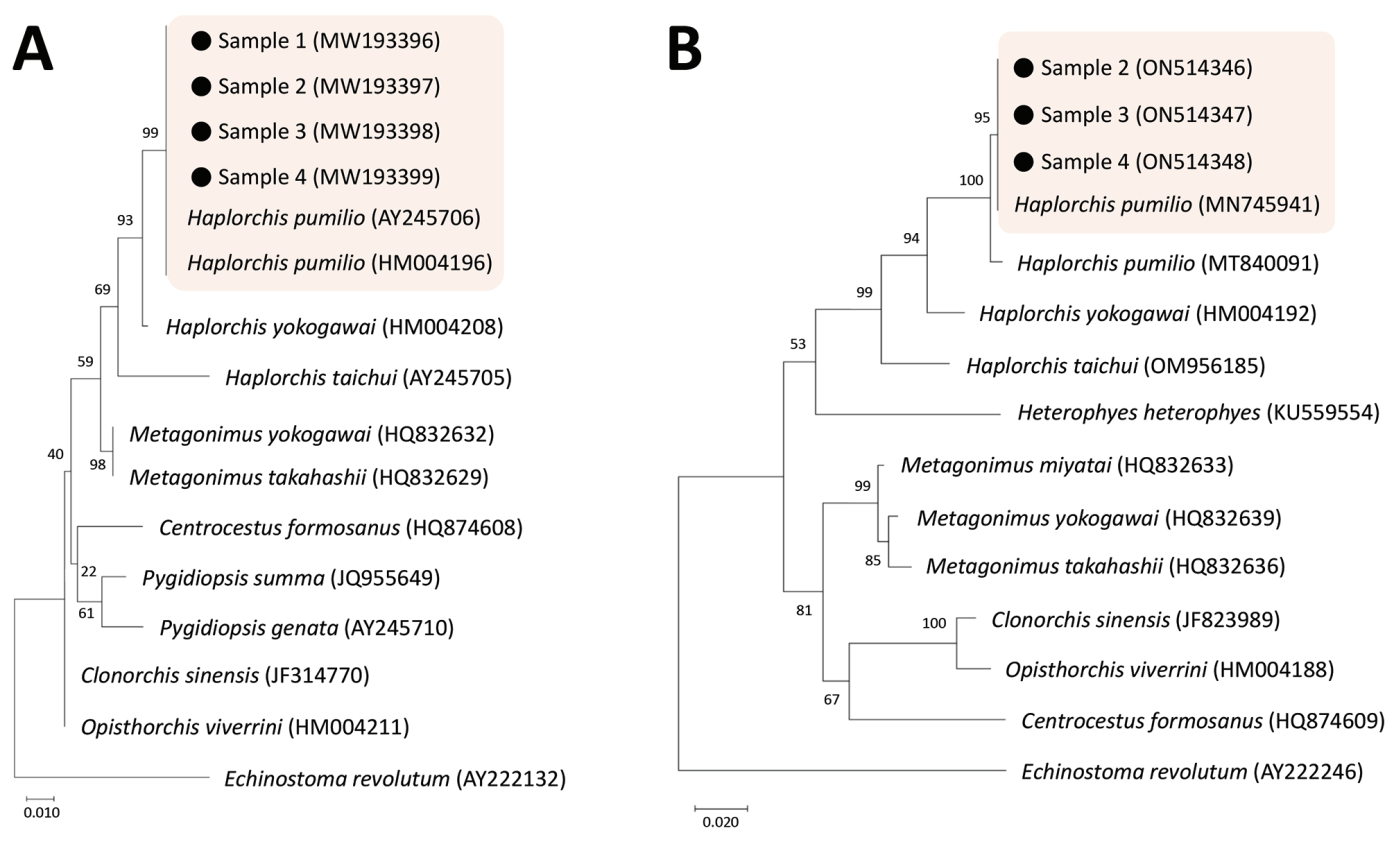
Phylogenetic trees of DNA of small trematode eggs from schoolchildren, Kome Island, Lake Victoria, Tanzania, in comparison with reference sequences of heterophyid (*Haplorchis pumilio* and others) and opisthorchiid trematodes, based on 18S (A) and 28S rDNA (B) sequences. The trees were constructed using the maximum-likelihood method based on the Kimura 2-parameter model and viewed by the MEGA 7.0 program (http://www.megasoftware.net). GenBank accession numbers are indicated. Scale bars indicate nucleotide substitutions per site.

Sequences of the 18S region of our samples (n = 4) were 100% identical to the 18S rDNA gene of *H. pumilio* in GenBank (accession nos. AY245706 and HM004196) (Table 2; Figure 2, panel A). However, only 95.0% identity was found between our samples and *Haplorchis taichui* (accession no. AY245705) and 98.9% was found between our samples and *Haplorchis yokogawai* (accession no. HM004208). However, using this gene, comparing our samples with *Heterophyes heterophyes* was not possible because 18S rDNA sequences of *H. heterophyes* are not available in GenBank.

**Table 2:** Sequence comparison of samples from study of *Haplorchis pumilio* eggs in schoolchildren, Kome Island, Lake Victoria, Tanzania, with other heterophyid and opisthorchiid flukes in GenBank based on 18S and 28S rDNA genes

18S rDNA	% Identity	28S rDNA	% Identity
Among study samples (*Haplorchis pumilio*), n = 4	100	Among study samples (*H. pumilio*), n = 3	100
*H. pumilio* (AY245706, Israel)	100	*Haplorchis pumilio* (MN745941, Kenya)	100
*H. pumilio* (HM004196, Thailand)	100	*Haplorchis pumilio* (MT840091, Brazil)	99.1
*Haplorchis yokogawai* (HM004208, Thailand)	98.9	*Haplorchis yokogawai* (HM004192, Thailand)	95.8
*Metagonimus yokogawai* (HQ832632, Japan)	97.2	*Haplorchis taichui* (OM956185, Vietnam)	93.7
*Metagonimus takahashii* (HQ832629, Japan)	97.2	*Metagonimus miyatai* (HQ832633, Japan)	91.4
*Pygidiopsis genata* (AY245710, Israel)	96.3	*Metagonimus yokogawai* (HQ832639, Japan)	91.4
*Clonorchis sinensis* (JF314770, China)	96.3	*Metagonimus takahashii* (HQ832636, Japan)	91.0
*Opisthorchis viverrine* (HM004211, Thailand)	96.3	*Heterophyes heterophyes* (KU559554, Italy)	86.9
*Centrocestus formosanus* (HQ874608, Thailand)	95.4	*Clonorchis sinensis* (JF823989, Vietnam)	89.3
*Pygidiopsis summa* (JQ955649, Korea)	95.2	*Opisthorchis viverrine* (HM004188, Thailand)	88.3
*Haplorchis taichui* (AY245705, Japan)	95.0	*Centrocestus formosanus* (HQ874609, Thailand)	88.2

Sequences of the 28S region of our samples (n = 3) were 99.1%–100% identical to the 28S rDNA gene of *H. pumilio* in GenBank (accession nos. MN745941 and MT840091) (Table 2; Figure 2, panel B). However, only 93.7% identity was found between our samples and *H. taichui* (accession no. OM956185) and 95.8% identity was found between our samples and *H. yokogawai* (accession no. HM004192). *H. heterophyes* (accession no. KU559554) appeared to be far from our samples (Figure 2, panel B) showing a sequence identity of only 86.9% (Table 2). Thus, we could confirm that our samples were mostly the eggs of *H. pumilio* and that Kome Island is a low-grade endemic area of *H. pumilio* infection among schoolchildren. However, possibilities remain for mixed infections with other heterophyid species (low worm loads and not detected by PCR).

## Conclusions

Taxonomically, in the genus *Haplorchis*, a total of 9 species have been known to be valid (*1*). Among them, 4 species are recognized to be zoonotic: *H. pumilio, H. taichui, H. yokogawai,* and *H. vanissimus* (*1*). Natural human infection with *H. pumilio* flukes was first documented in Egypt in 1977 in a 9-year-old child passing diarrheic stools (*4*). A vital snail species for *H. pumilio* flukes is *Melanoides tuberculata* in Egypt, Taiwan, India, Peru, and Brazil (*1*,*5*). Their metacercariae are detected in various species of freshwater or brackish water fish, including *Mugil* sp., *Tilapia* sp., and *Bagrus bayad* (*1*,*6*).

In Africa, with the exception of Egypt (an *H. pumilio* fluke–endemic area), the distribution of *H. pumilio* flukes has been rarely reported. In Kenya, *H. pumilio* cercariae were confirmed molecularly recently in *M. tuberculata* snails; a high positive rate of 69.4% was found in the northernmost area of Lake Victoria, in Kenya (*7*). However, human *H. pumilio* infection in sub-Saharan Africa countries, including Kenya, has not been reported. In São Tomé and Principe, a sub-Sahara country off the west coast of Africa, eggs of Heterophyidae, which are very similar to *Metagonimus yokogawai*, were found in 28.2% of 1,050 human fecal samples in 1987, but their species could not be identified (*8*). Those eggs were 22.2–27.7 × 17.0–21.0 μm in size and had a thick wall and a difficult-to-see operculum (*8*); they were markedly different from the eggs of *Haplorchis* or *Heterophyes* spp (*1*). Of note, a zoonotic liver fluke species, *Opisthorchis felineus*, was found in dogs and cats in New Bussa, Nigeria (*9*); however, this species has never been found to distribute around Lake Victoria.

On the Lake Victoria basin, schistosomiasis and soil-transmitted helminthiases have been acknowledged as major public health problems (*10*), whereas intestinal fluke infections have been poorly studied. In this study, we detected a low-grade endemicity of *H. pumilio* infection on Kome Island, Lake Victoria, Tanzania. It remains unclear if human *H. pumilio* infection has been endemic on Kome Island unnoticed for a long time or was introduced recently. These 2 possibilities should be investigated.

Surveyed schoolchildren on Kome Island had no history of international travel, including to Asia, South America, Egypt, and Kenya, where the *H. pumilio* fluke is endemic. Therefore, the source of infection in our cases seems to be the fish host caught around Kome Island. In Lake Victoria, 3 fish species are known to predominate, and one of them is Nile tilapia (*Oreochromis niloticus*), which is a fish host for *H. pumilio* flukes (*1*,*11*,*12*). Nile tilapia is popularly eaten on Kome Island and is highly suggested as the source of infection in our cases. Studies are required to determine the existence of the life cycle of *H. pumilio* flukes on and around Kome Island and clarify the public health importance of *H. pumilio* infection in this area.
